# The relationship between bioelectrical impedance phase angle and subjective global assessment in advanced colorectal cancer

**DOI:** 10.1186/1475-2891-7-19

**Published:** 2008-06-30

**Authors:** Digant Gupta, Christopher G Lis, Sadie L Dahlk, Jessica King, Pankaj G Vashi, James F Grutsch, Carolyn A Lammersfeld

**Affiliations:** 1 Cancer Treatment Centers of America^® ^(CTCA) at Midwestern Regional Medical Center, 2610 Sheridan Road, Zion, IL, 60099, USA

## Abstract

**Background:**

Bioelectrical Impedance (BIA) derived phase angle is increasingly being used as an objective indicator of nutritional status in advanced cancer. Subjective Global Assessment (SGA) is a subjective method of nutritional status. The objective of this study was to investigate the association between BIA derived phase angle and SGA in advanced colorectal cancer.

**Methods:**

We evaluated a case series of 73 stages III and IV colorectal cancer patients. Patients were classified as either well-nourished or malnourished using the SGA. BIA was conducted on all patients and phase angle was calculated. The correlation between phase angle and SGA was studied using Spearman correlation coefficient. Receiver Operating Characteristic curves were estimated using the non-parametric method to determine the optimal cut-off levels of phase angle.

**Results:**

Well-nourished patients had a statistically significantly higher (p = 0.005) median phase angle score (6.12) as compared to those who were malnourished (5.18). The Spearman rank correlation coefficient between phase angle and SGA was found to be 0.33 (p = 0.004), suggesting better nutritional status with higher phase angle scores.

A phase angle cut-off of 5.2 was 51.7% sensitive and 79.5% specific whereas a cut-off of 6.0 was 82.8% sensitive and 54.5% specific in detecting malnutrition. Interestingly, a phase angle cut-off of 5.9 demonstrated high diagnostic accuracy in males who had failed primary treatment for advanced colorectal cancer.

**Conclusion:**

Our study suggests that bioimpedance phase angle is a potential nutritional indicator in advanced colorectal cancer. Further research is needed to elucidate the optimal cut-off levels of phase angle that can be incorporated into the oncology clinic for better nutritional evaluation and management.

## Background

Bioelectrical Impedance Analysis (BIA) is an objective, easy-to-use, quick, non-invasive, and reproducible technique to evaluate changes in body composition. BIA is increasingly being used to assess nutritional status in patients with cancer [1-7]. BIA measures body component resistance (R) and reactance (Xc) by recording a voltage drop in applied current [8]. Reactance causes the current to lag behind the voltage creating a phase shift. This shift is quantified geometrically as the angular transformation of the ratio of reactance to resistance, or the phase angle [9].

Phase angle reflects the relative contributions of fluid (resistance) and cellular membranes (reactance) of the human body and has been suggested to be an indicator of body cell mass and nutritional status [1]. By definition, phase angle is positively associated with reactance and negatively associated with resistance [9]. Lower phase angles suggest cell death or decreased cell integrity, while higher phase angles suggest large quantities of intact cell membranes [10]. Phase angle has been found to be a prognostic indicator in several clinical conditions such as human immunodeficiency virus infection, liver cirrhosis, chronic obstructive pulmonary disease, hemodialysis, sepsis, lung cancer colorectal cancer, and pancreatic cancer [7,10-17]. For the lack of a well-agreed upon cut off level for phase angle, previously published studies have utilized either the mean or the median phase angle scores of their respective patient populations to predict survival. Although the cut-off levels for phase angle suggested by the above studies seem to be in agreement with each other, there is a clear need to define optimal thresholds of phase angle as an indicator of nutritional status in advanced cancer.

Subjective Global Assessment (SGA) is a subjective, simple, safe, inexpensive and effective method to assess nutritional status in advanced cancer [1]. The SGA is a clinical technique that combines data from subjective and objective aspects of medical history (weight change, dietary intake change, gastrointestinal symptoms, and changes in functional capacity) and physical examination (low levels of subcutaneous fat and muscle mass, ankle or sacral edema and ascites) [18]. After evaluation, patients are categorized into three distinct classes of nutritional status; well-nourished (SGA A), moderately malnourished (SGA B) and severely malnourished (SGA C). The SGA has been extensively validated as a nutritional assessment technique in oncology patients [1,19,20].

The objective of this study was to investigate the association between BIA derived phase angle (an objective method of nutritional assessment) and SGA (a subjective method of nutritional assessment) in advanced colorectal cancer.

## Methods

### Patients

A retrospective chart review was performed on a consecutive case series of 73 stages III and IV colorectal cancer patients treated at Cancer Treatment Centers of America (CTCA) at Midwestern Regional Medical Center (MRMC) between January 2000 and March 2003. The patients were identified from the MRMC tumor registry. All patients had a histologically confirmed diagnosis of stages III and IV colorectal cancer. All tumors were adenocarcinomas. Tables 1 and 2 show the baseline characteristics of our patient cohort. The study was approved by the Institutional Review Board at Midwestern Regional Medical Center.

**Table 1:** Baseline Characteristics (N = 73)

Characteristic	Number	Percent (%)
Sex		
Male	41	50.6
Female	40	49.4

Prior Treatment History		
Progressive disease	42	51.9
Newly diagnosed	39	48.1

Tumor Stage at Diagnosis		
Stage III	29	35.8
Stage IV	52	64.2

Tumor Grade at Diagnosis		
Well	3	3.7
Moderate	56	69.1
Poor	18	22.2
Unknown	4	4.9

Subjective Global Assessment		
Well-nourished	44	60.3
Malnourished		
*Moderately malnourished*	23	31.5
*Severely Malnourished*	6	8.2

**Table 2:** Baseline Characteristics (N = 73)

Characteristic	Mean	Standard Deviation	Range	Normal Values
Age at diagnosis (years)	56	11.4	29 – 82	
Albumin (g/dl)	3.6	0.47	2.2 – 4.7	3.4 – 5.4
Phase Angle (degrees)	5.7	1.3	3.2 – 10.7	3 – 10
Prealbumin (mg/dl)	21.2	7.4	8.0 – 38	15.7 – 29.6
Transferrin (mg/dl)	244.7	57.3	76 – 397	250 – 300

g = grams

mg = milligrams

dl – deciliter

### Nutritional assessment

All patients underwent a baseline nutritional assessment, which included SGA and BIA. SGA was performed by registered dietitians who reviewed the SGA instrument with the patient to obtain answers to all the questions. The dietitians also completed a physical exam paying particular attention to low levels of subcutaneous fat and muscle mass, presence of ankle and sacral edema and ascites. After the consultation, the dietitians ranked the patient's nutritional status as well-nourished (SGA A), moderately malnourished (SGA B) or severely malnourished (SGA C). For the purpose of this analysis, malnutrition was defined as either SGA B or SGA C. BIA was performed using a Bioelectrical Impedance Analyzer, Model BIA-101Q: RJL Systems, Clinton Township, MI, USA. BIA was conducted while patients were lying supine on a bed or exam table, with legs apart and arms not touching the torso. All evaluations were conducted on the patients' right side using the four surface standard electrode (tetra polar) technique on the hand and foot [21]. Resistance (R) and reactance (Xc) were directly measured in Ohms at 50 kHz, 800 μA using RJL BIA. One assessment of resistance (R) and reactance (Xc) was made. Phase angle was calculated using the following equation: Phase Angle = (Resistance/Reactance)*(180/π).

### Statistical analysis

All data were analyzed using SPSS 11.5 (SPSS Inc., Chicago, IL, USA). For the purpose of this analysis, patients were classified as either well-nourished (SGA-A) or malnourished (SGA-B and SGA-C). The SGA-B and SGA-C were merged together because of only 6 observations for SGA-C. Phase angle was found to be nonnormally distributed as demonstrated by the Shapiro Wilk test statistic. The median phase angle scores were compared across the 2 categories of nutritional status using non-parametric Mann Whitney test. The correlation between phase angle and SGA was studied using Spearman correlation coefficient owing to nonnormal distribution of phase angle. Receiver Operating Characteristic (ROC) curves were estimated using the non-parametric method [22,23] to further evaluate the association between phase angle as an indicator of nutritional assessment and SGA. The area under the curve (AUC) was calculated to determine the accuracy of phase angle as a nutritional assessment tool. The further the curve lies above the reference line, the more accurate the test. Coordinates of the curve were examined across the full range of potential phase angle cut-off values in an attempt to select an optimal phase angle cut-off that properly balanced the needs of sensitivity and specificity. Since smaller values of phase angle are believed to indicate worsening of nutritional status, sensitivity was defined as the proportion of malnourished patients with phase angle results smaller than the cut-off, i.e. the ability of phase angle cut-off to estimate truly malnourished patients. Similarly, specificity was defined as the proportion of well-nourished patients with phase angle results greater than equal to the cut-off, i.e. the ability of phase angle cut-off to estimate truly well-nourished patients.

## Results

Tables 1 and 2 show the baseline characteristics of our patient sample.

The distribution of phase angle scores across the two classes of nutritional status using non-parametric Mann Whitney test found that well-nourished patients had a statistically significantly higher (p = 0.005) median phase angle score (6.12) as compared to those who were malnourished (5.18). The Spearman rank correlation coefficient between phase angle and SGA was found to be 0.33 (p = 0.004), suggesting better nutritional status with higher phase angle scores.

**Figure 1:**
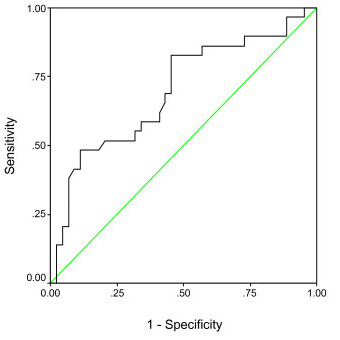
A receiver operating characteristic curve assessing an optimal cut-off point of phase angle as a marker for malnutrition as defined by the SGA (N = 73).

Figure 1 shows the ROC curve for phase angle. The curve reveals that phase angle provides modest diagnostic accuracy to distinguish between well-nourished and malnourished status (AUC = 0.7; 95% CI = 0.57 to 0.82, p = 0.005). The sensitivities and specificities of potential phase angle cut-offs, as suggested by the coordinates of the curve data, are shown in Table 3. It was difficult to identify an optimal cut-off level of phase angle with simultaneously high levels of sensitivity and specificity. The table suggests that the phase angle cut-off value of 5.7 combines modest levels of sensitivity (69%) with low levels of specificity (56.8%). Increasing the phase angle cut-off to 6.0 raises the sensitivity level to 82.8% without much loss in the level of specificity (54.5%). This implies that using a cut-off level of 6.0 for phase angle, 82.8% of truly malnourished patients will be correctly identified as malnourished, whereas 45.5% (1-specificity) of truly well-nourished patients will be incorrectly identified as malnourished. On the other hand, using 5.2 as the cut-off value for phase angle, only 51.7% of truly malnourished patients will be correctly identified as malnourished, whereas 20.5% (1-specificity) of truly well-nourished patients will be incorrectly identified as malnourished.

**Table 3:** Sensitivities and specificities of selected phase angle cut-off levels (N = 73)

Phase Angle Cut-off	Sensitivity (%)	Specificity (%)
5.2	51.7	79.5
5.3	55.2	68.2
5.4	58.6	65.9
5.7	69.0	56.8
6.0	82.8	54.5

**Figure 2:**
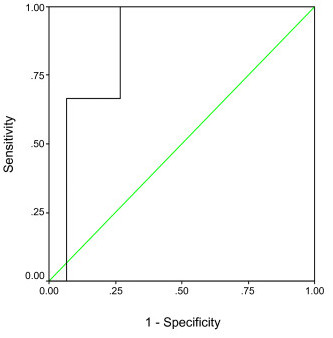
A receiver operating characteristic curve assessing an optimal cut-off point of phase angle as a marker for malnutrition as defined by the SGA in males with progressive colorectal cancer (N = 18).

In order to examine the differences due to gender and prior treatment history, separate ROC curves were constructed for males, females, newly diagnosed patients and patients with prior treatment history. ROC curves were also constructed for different combinations of gender and prior treatment history. The area under the curve, optimal phase angle cut-off levels, and the corresponding sensitivities and specificities for all patient subgroups are displayed in Table 4. The only patient subgroup for which high levels of both sensitivity and specificity could be obtained was males with progressive disease. In this patient subgroup, a phase angle cut-off level of 5.9 was 100% sensitive and 73.3% specific in diagnosing malnutrition. Figure 2 shows the ROC curve for phase angle in this patient subgroup.

**Table 4:** Receiver Operating Characteristic analysis based on gender and prior treatment history

Patient Subgroup	N	AUC (95% CI)	Cut-off level	Sensitivity (%)	Specificity (%)
Males	38	0.67 (0.48 – 0.85)	6.0	73.3	65.2
Females	35	0.75 (0.58 – 0.92)	5.6	71.4	47.6
Newly Diagnosed	33	0.64 (0.45 – 0.83)	6.0	72.2	46.7
Progressive Disease	40	0.79 (0.65 – 0.93)	5.6	81.8	62.1
Males with Progressive Disease	18	0.87 (0.69 – 1.0)	5.9	100	73.3
Females with Progressive Disease	22	0.72 (0.50 – 0.95)	6.0	100	42.9

N = Sample Size

AUC = Area Under the Curve

CI = Confidence Interval

## Discussion

Although BIA derived phase angle has been used as a complementary nutritional assessment tool in cancer, we need to be able to choose a specific cut-off level that can help the treating oncologists and clinical nutritionists classify cancer patients as either well-nourished or malnourished. The choice of the cutoff is mandated by the need to closely match the sensitivity and specificity of the traditional nutritional tests. One way of achieving this goal is to evaluate phase angle against a test that has been extensively validated in similar treatment settings. This study was undertaken to investigate the association between BIA derived phase angle as an indicator of nutritional status, and SGA in advanced colorectal cancer.

The sensitivities and specificities considered together for potential optimal cut-off levels of phase angle were found to be modest at best and the test was found to be either too sensitive or too specific. We found that using different cut-off levels for males versus females, and for newly diagnosed patients versus those with progressive disease might be more appropriate as opposed to using single cut-off level for all patients. Interestingly, a phase angle cut-off of 5.9 demonstrated high diagnostic accuracy in males who had failed primary treatment for advanced colorectal cancer. Our findings are consistent with those reported by another group of researchers who evaluated phase angle against SGA in 279 patients undergoing elective gastrointestinal surgery [1]. The study found a fair overall agreement between SGA and BIA estimates and couldn't obtain an optimal phase angle cut-off with high sensitivity and specificity. The study also suggested different potential cut-offs for men (6.3) and women (5.9) indicating a better balance of sensitivity and specificity. A study conducted in patients with advanced lung cancer stratified the patient cohort by the mean phase angle score of 4.5. Interestingly, patients with phase angle scores less than or equal to 4.5 had a significantly shorter survival than those with phase angle scores greater than 4.5 [7]. Another study conducted in HIV-infected patients stratified patients into 4 quartiles, with 5.3, 5.9 and 6.5 as the cut-off points. The study found phase angle to be an independent prognostic marker of clinical progression and survival [17]. In another prospective study of liver cirrhosis patients, phase angle equal to or less than 5.4 was associated with shorter survival as compared to phase angle greater than 5.4 [10].

In our study, no optimal phase angle cut-off level with simultaneously high levels of sensitivity and specificity could be identified. There are several potential explanations of these findings. 1. BIA derived phase angle is not a valid indicator of nutritional status in advanced cancer, 2. Phase angle and SGA capture different aspects of nutritional status and might complement each other in overall nutritional evaluation, 3. Phase angle is a valid marker of nutritional status and the relatively modest correlations observed in the present study might be escalated using a larger sample size. In the present study a phase angle cut-off level of 5.2 had low sensitivity but high specificity whereas a cut-off level of 6.0 had high sensitivity but low specificity. It is likely that an optimal phase angle cut-off level is located somewhere between these two values. We believe that the goal of achieving an optimal phase angle cut-off with high levels of sensitivity and specificity should be further explored in similar patient populations with larger sample sizes.

ROC analysis at best provides guidelines for which cut-offs should be considered. We believe that the choice of an optimal cut-off level for any diagnostic test is context dependent. In our study, we evaluated the optimal cut-off levels of phase angle as a nutritional assessment tool in advanced colorectal cancer. Since malnutrition is a major cause of morbidity and mortality in these patients, the treating oncologists and clinical nutritionists might find it more worthwhile to be able to correctly identify a high proportion of malnourished patients (a high sensitivity) even though it comes at the expense of reduced specificity (a high rate of false positives). In such situations, selecting a high cut-off level of 6 makes more sense as opposed to selecting a low cut-off level of 5.2.

What exactly is phase angle? Some earlier studies have tried to address these questions, albeit in a limited capacity. For instance, Schwenk et al. hypothesized that phase angle could possibly be interpreted as a global marker of malnutrition in HIV infected patients [17]. In another study conducted on HIV-infected patients, it was argued that phase angle reflects the integrity of vital cell membranes [15]. In patients with liver cirrhosis, phase angle was speculated to be a marker of clinically relevant malnutrition characterized by both increased extracellular mass and decreased body cellular mass [10]. In advanced lung cancer, phase angle was speculated to be an indicator of altered tissue electrical properties [7].

Limitations of this study relate to the BIA technique, retrospective study design and small sample size. This study, because of its retrospective nature, relies on data not primarily meant for research. The subgroup ROC analyses were based on small sample sizes without accounting for the number of multiple comparisons made in this study. The non-normal distribution of phase angle in our study could be an effect of the small sample size. Despite these limitations, our study provides valuable insights on what might be an appropriate phase angle cut-of level in patients with advanced colorectal cancer. Clearly, there is a need to validate the diagnostic accuracy of phase angle using larger sample sizes in advanced cancer populations.

It has been suggested that the variability of direct bioimpedance measures (resistance, reactance, and phase angle) depends on age, gender, and body mass characteristics of the study population which could possibly limit the extrapolation of the model [24,25]. Some other reported limitations of using BIA for assessment of body composition are hydration status and/or major disturbances of water distribution, body position during procedure, ambient air and skin temperatures, recent physical activity, conductance of the examining table, abstinence from alcohol and caffeinated beverages, food consumption and voiding 30 minutes prior to measurement [26]. Since the original intent of the BIA in this study was to gather estimates of body composition as part of a baseline nutritional assessment in a clinical setting, not all of these factors could realistically be controlled. Patients were free of visible edema or ascites so there was control for obvious overhydration. However, it is to be noted that hydration status in cancer patients are often due to treatment, therefore, just observing obvious signs of hydration may have overlooked changes that could further have influenced measurement validity. Body position was controlled for because all patients were in the supine position in a bed or on an exam table. Air temperature was within a controlled range in our hospital setting. Physical activity was limited in these patients due to the advanced nature of their disease. Food intake was not controlled for in this clinical setting, which may have contributed to a small amount of variability. No assessment of inter-rater reliability of the users of BIA and SGA was made in this study. This bias, however, was minimized by restricting the use of BIA and SGA to well-trained dietitians with an expertise in the use of these clinical techniques. Moreover, BIA was conducted in all patients using the same analyzer. Finally, we believe that future studies should use a more objective method of nutritional assessment in order to derive the definitive cut-offs of phase angle.

## Conclusion

In summary, our study suggests that bioimpedance phase angle is a potential indicator of nutritional status in advanced colorectal cancer. Further research is needed to elucidate the optimal cut-off levels of phase angle that can be incorporated into the oncology clinic for better nutritional evaluation and management.

## Competing interests

The authors declare that they have no competing interests.
